# rs4919510 in *hsa-mir-608* Is Associated with Outcome but Not Risk of Colorectal Cancer

**DOI:** 10.1371/journal.pone.0036306

**Published:** 2012-05-11

**Authors:** Bríd M. Ryan, Andrew C. McClary, Nicola Valeri, Dillon Robinson, Alessio Paone, Elise D. Bowman, Ana I. Robles, Carlo Croce, Curtis C. Harris

**Affiliations:** 1 Laboratory of Human Carcinogenesis, Center for Cancer Research, National Cancer Institute, Bethesda, Maryland, United States of America; 2 Howard Hughes Medical Institute, National Cancer Institute, Bethesda, Maryland, United States of America; 3 Department of Molecular Virology, Immunology and Medical Genetics, Comprehensive Cancer Center, Ohio State University, Columbus, Ohio, United States of America; IFOM, Fondazione Istituto FIRC di Oncologia Molecolare, Italy

## Abstract

**Background:**

Colorectal cancer is the third most incident cancer and cause of cancer-related death in the United States. MicroRNAs, a class of small non-coding RNAs, have been implicated in the pathogenesis and prognosis of colorectal cancer, although few studies have examined the relationship between germline mutation in the microRNAs with risk and prognosis. We therefore investigated the association between a SNP in *hsa-mir-608*, which lies within the 10q24 locus, and colorectal cancer.

**Methods and Results:**

A cohort consisting of 245 cases and 446 controls was genotyped for rs4919510. The frequency of the GG genotype was significantly higher in African Americans (15%) compared to Caucasians (3%) controls. There was no significant association between rs4919510 and colorectal cancer risk (African American: OR_GG *vs.* CC_ 0.89 [95% CI, 0.41–1.80]) (Caucasian: OR_GG *vs.* CC_ 1.76, ([95% CI, 0.48–6.39]). However, we did observe an association with survival. The GG genotype was associated with an increased risk of death in Caucasians (HR_GG *vs.* CC_ 3.54 ([95% CI, 1.38–9.12]) and with a reduced risk of death in African Americans (HR_GG *vs.* CC_ 0.36 ([95% CI 0.12–1.07).

**Conclusions:**

These results suggest that rs4910510 may be associated with colorectal cancer survival in a manner that is dependent on race.

## Introduction

Colorectal carcinoma is the third most incident cancer and cause of cancer-related mortality in the United States. Estimates for 2011 predict 141,210 new cases of colorectal cancer and 49,380 deaths [1]. In the past ten years, there have been significant advances in our understanding of the natural history and molecular mechanisms underlying colorectal cancer development. In addition, substantive guidelines for screening have been developed by the US Preventative Services Task Force. Therapeutic improvements have also been achieved, particularly after the 2004 approval of bevacizumab (Avastin®) for advanced disease. Despite these advances, the five-year survival for colorectal cancer has increased only ∼5 percentage points in recent years [1].

Although this survival trend holds true for both African Americans and Caucasians, a readily apparent health disparity exists for African Americans. By age 65, African Americans have an approximate 25% greater probability of developing colorectal carcinoma compared to Caucasians [1], [2]. Additionally, colorectal cancer incidence in African Americans in 2007 was estimated to be essentially the same as in 1975, whereas Caucasians have experienced a 17-percentage point drop in incidence. Twenty four percent of colorectal cancers are detected at late stage in African Americans, compared to 19% in Caucasians. This may in part explain the significantly worse overall survival rate in African Americans, who have a 15% increased risk of dying from colorectal cancer when compared to Caucasians [1], [2].

MicroRNAs (miRNAs) are a class of small, non-coding RNAs consisting of approximately 22–25 nucleotides in their processed, mature form. To date, over 1000 miRNAs have been discovered in humans, with some estimates predicting a final count of several thousand [3], [4], Physiologically, miRNAs act as a rheostat, fine tuning translational output through targeted mRNA binding and repression. The target repertoire of a miRNA is defined primarily by its *seed* sequence, nucleotides 2–6 at the 3′ end of its mature form. A variety of pathologic associations have been attributed to altered miRNA networks particularly in cancer, with miRNAs able to function as both oncogenes and tumor suppressors [5], [6], [7].

Numerous studies have linked both aberrant expression and genetic variation in miRNAs to colorectal cancer risk, diagnosis, prognosis, and drug response [8], [9], [10], [11]. Single nucleotide polymorphisms (SNPs) in miRNAs can affect their biogenesis, processing, and/or target site binding in a variety of ways, as highlighted in our recent review [12]. For example, since microRNAs are processed in a step-wise fashion from *pri* to *pre* to a mature strand, a process guided by stereotypical secondary structure, one can conceptualize how a single base pair change could affect processing or recognition by guide components [12]. Alternatively, a SNP in the mature sequence can alter target site interactions by either strengthening or weakening hybridization kinetics, or if in the seed sequence, it can significantly transform the target library of the miRNA itself. Interestingly, the prevalence of these SNPs in miRNAs is significantly lower than predicted in the remainder of the genome, speaking to the evolutionary conservation and importance of these small biomolecules [13], [14]. However, a few reports have recently highlighted an association between these low frequency germline variations and cancer risk and/or prognosis [12].

**Table 1:** Demographic characteristics of the NCI-University of Maryland colorectal cancer case control study participants.

Characteristics	Cases	Population Controls	Hospital Controls	Total Controls
**n**	245	236	210	446
**Age ± SD**	64.7±11.7	66.8±9.7	63.1±12.1	65.0±11.0
**Gender (%)**				
Male	185 (75)^1^	115 (48)	97 (46)	212 (48)
Female	60 (25)	121 (51)	113 (54)	234 (52)
**Race (%)**				
African American	97 (40)	121 (51)	69 (33)	190 (43)
Caucasian	148 (60)	115 (49)	141 (67)	256 (57)
**Stage (%)**				
Unknown	4 (31)			
I	38 (16)			
II	69 (29)			
III	79 (33)			
IV	51 (21)			
**Survival Time (months)**				
Median, range	50.5 (43.2–56.4)			

1 Statistically significant; *p*-value<0.05 vs. each control group.

Loss of heterozygosity in the 10q24 locus has been reported in a number of human cancers, including but not limited to colorectal, prostate, pancreatic, and brain [15], [16], [17], [18]. Despite numerous investigations into a possible tumor suppressor gene in this hotspot, strong evidence is lacking [19]. Interestingly, *hsa-mir-608*, a microRNA of which virtually nothing is known functionally, lies within an intron of *SEMA4G* in this region. Furthermore, *hsa-mir-608* harbors a SNP, rs4919510, in bp 22 of its mature 25 bp sequence. This C-G polymorphism is common in several populations. Here we report that the rs4919510 germline polymorphism within *hsa-mir-608* is associated with colorectal cancer survival, in a race-specific manner.

**Table 2:** Association of rs4919510, a SNP in *mir-608*, with colorectal cancer risk.

Stratification	Genotype	Cases N (%)	Controls N (%)	Univariable OR (95% CI)	*p*-value	Multivariable^2^ OR (95% CI)	*p*-value
All	CC	124 (52)	231 (53)	Reference	0.743	Reference	0.183
	CG	96 (40)	166 (38)	1.06 (0.75–1.48)	0.393	1.28 (0.89–1.84)	0.910
	GG	19 (8)	36 (8)	0.78 (0.43–1.39)	0.831	0.96 (0.51–1.82)	0.521
	p-trend			1.03 (0.80–1.31)		1.09 (0.84–1.43)	
African Americans	CC	34 (36)	62 (34)	Reference	0.550	Reference	0.662
	CG	48 (51)	95 (51)	1.17 (0.69–1.98)	0.689	1.13 (0.66–1.93)	0.619
	GG	12 (13)	28 (15)	0.86 (0.41–1.80)	0.553	0.83 (0.39–1.78)	0.795
	p-trend			0.89 (0.62–1.30)		0.95 (0.66–1.37)	
Caucasians	CC	90 (62)	169 (68)	Reference	0.221	Reference	0.125
	CG	48 (33)	71 (29)	1.36 (0.83–2.23)	0.393	1.49 (0.89–2.49)	0.622
	GG	7 (5)	8 (32)	1.76 (0.48–6.39)	0.190	1.40 (0.37–5.27)	0.145
	p-trend			1.27 (0.89–1.83)		1.38 (0.89–2.13)	

2 Adjusted for age, gender, and race (where applicable).

**Table 3:** Association of rs4919510 with colorectal cancer survival.

Stratification	Genotype	Alive N (%)	Deceased N (%)	Univariable HR (95% CI)	*p*-value	Multivariable^3^ HR (95% CI)	*p*-value
All	CC	66 (53)	58 (50)	Reference	0.764	Reference	0.794
	CG	48 (39)	48 (42)	1.06 (0.72–1.56)	0.883	1.06 (0.70–1.60)	0.581
	GG	10 (8)	9 (8)	1.05 (0.52–2.13)	0.730	1.23 (0.59–2.60)	0.608
	p-trend			1.04 (0.78–1.39)		1.09 (0.79–1.49)	
African Americans	CC	13 (30)	21 (42)	Reference	0.243	Reference	0.271
	CG	23 (52)	25 (50)	0.70 (0.39–1.27)	0.082	0.72 (0.40–1.30)	0.066
	GG	9 (18)	4 (8)	0.38 (0.13–1.13)	0.059	0.36 (0.12–1.07)	0.180
	p-trend			0.65 (0.42–1.02)		0.72 (0.45–1.16)	
Caucasians	CC	53 (66)	37 (57)	Reference	0.406	Reference	0.220
	CG	25 (31)	23 (35)	1.25 (0.74–2.10)	**0.009**	1.41 (0.81–2.44)	**0.027**
	GG	2 (32)	5 (8)	**3.54 (1.38–9.12)**	**0.046**	**2.95 (1.13–7.71)**	**0.030**
	p-trend			**1.52 (1.01–2.31)**		**1.57 (1.05–2.37)**	

3 Adjusted for age, gender, race, and stage.

## Materials and Methods

### Ethics statement

All participants gave written informed consent. The protocol was in compliance with the Declaration of Helsinki and approved by the Institutional Review Boards of the National Cancer Institute. The ethics exemption number is 11289.

### Study Population: The NCI-University of Maryland Colorectal Cancer Case-Control Study

The study population consisted of 691 subjects. Incident colorectal cancer cases (n = 245) and controls (n = 446) were recruited from 1992–2003 and 1998–2003, respectively from the greater Baltimore, Maryland area. The controls were accrued from both a hospital setting (n = 236) and a community setting (n = 210). The inclusion and exclusion criteria have been previously described [20]. In brief, subjects were self-reported Caucasian or African-Americans born in the United States, and were excluded if they self-reported a history of cancer other than colorectal, HIV, HBV, HCV, or IV drug use, were institutionalized, or had a mental impairment. Information to determine disease stage, treatment, and survival was obtained from medical records and pathology reports, Social Security Death Index, and National Death Index. Disease staging was completed according to the tumor-node-metastasis system of the American Joint Committee on Cancer. The survival period was determined from date of hospital admission for surgery to date of last completed search for death entries in the Social Security Death Index (2010). Informed consent was obtained from all participants, and epidemiological questionnaires including, personal history, family medical history, past medical history, tobacco history, dietary information, and information on work environment, were administered to all subjects. The study was approved by the institutional review boards of the participating institutions.

**Figure 1:**
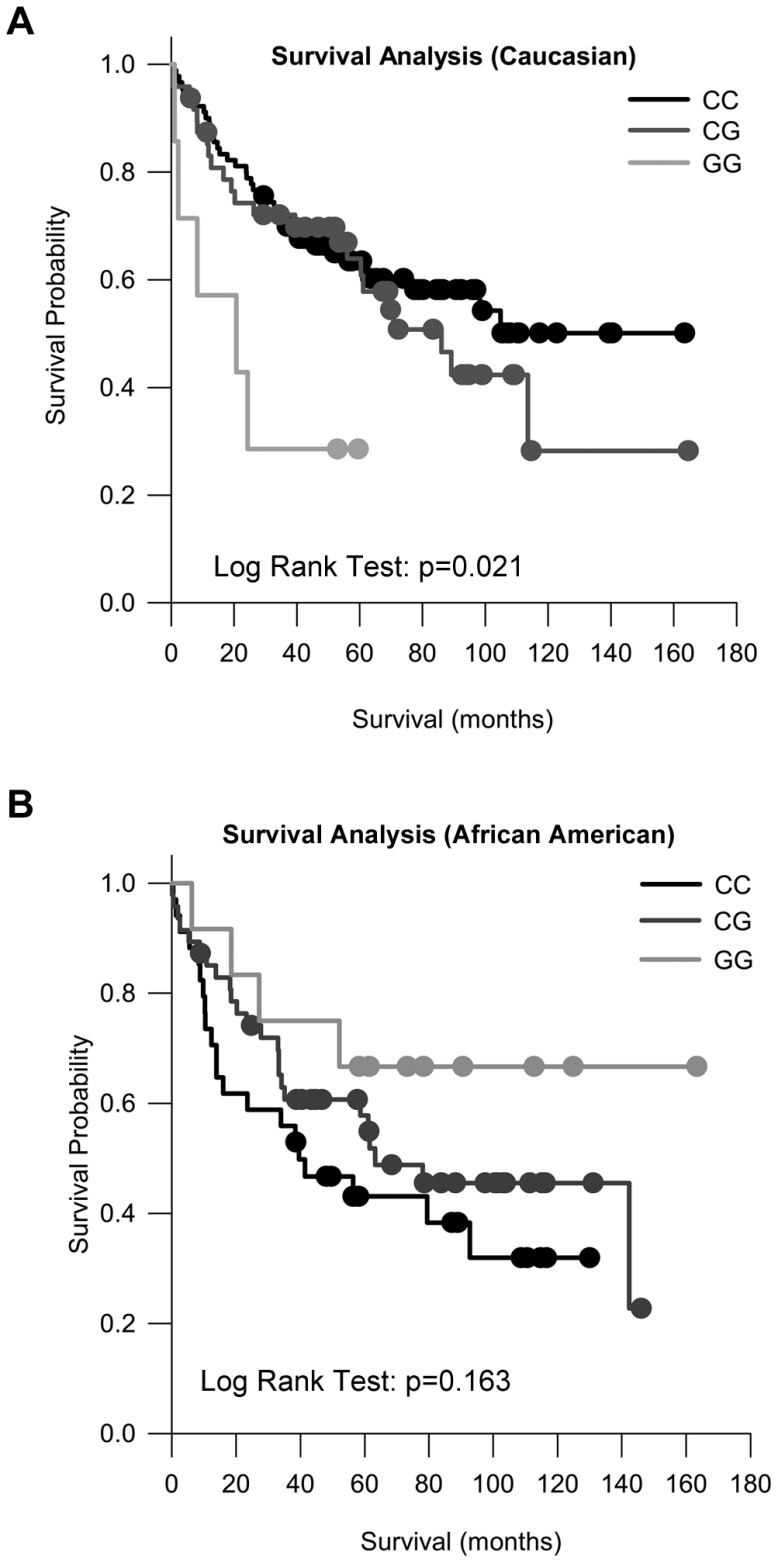
Rs4919510 and colorectal cancer survival stratified by race. Kaplan Meier survival curves depicting the relationship between rs4919510 and colorectal cancer survival in A) Caucasians (n = 145) and B) African Americans (n = 94). Censored individuals are indicated with a closed circle.

#### Genotyping

Genomic DNA was isolated from buffy coat or colorectal tissue using the Qiagen FlexiGene DNA Kit or the DNAeasy tissue kit, respectively (Qiagen, Valencia, CA). Cases and controls were genotyped using the Taqman assay (Life Technologies, Carlsbad, CA) for rs4919510 in *mir-608* at Ohio State University Genotyping Core. The case, control, negative controls, and duplicate samples were randomly distributed for order of processing, with 10% duplicates to test both inter- and intra-plate concordance. All parties involved in genotyping were blinded to the case, control, and duplicate status of the samples. Samples that failed to genotype were recorded as undetermined. Both inter- and intra-plate duplicates were 100% concordant. The completion rate was 98%.

#### KRAS mutation analysis

Samples with tumor tissue were sent for KRAS mutation analysis at Bioserve (BioServe Biotechnologies, Ltd. Beltsville, MD). DNA was extracted using Trizol™ according to the manufacturer's instructions. The following mutations were assessed: codon 12 (nucleotide 1; 0.8%), codon 12 (nucleotide 2; 20%), codon 13 (nucleotide 1; 2.3%) and codon 61 (nucleotide 3; 0.8%). Data in this study is represented as normal or mutant for any one of the mutations outlined above (26% overall).

#### Statistical Analysis

Statistical analyses were performed using STATA 11.0 (College Station, TX). A p-value of less than 0.05 was used as the criterion for statistical significance, and all statistical tests were two-sided. Departures from Hardy-Weinberg equilibrium were determined using a χ^2^ test. Odds ratios (OR) and their corresponding 95% confidence intervals (CI) were estimated using a univariable unconditional logistic regression model and an adjusted model that included the covariates age (continuous) and gender (categorical). Hazard ratios (HR) and 95% CI were estimated using a univariable Cox proportional hazards regression model and an adjusted model for the covariates age (continuous), gender (categorical) and stage of disease (categorical). Survival time was calculated from date of surgery to date of either last known follow up (last NDI update 12/31/2008) or date of death due to colorectal cancer. Of the 245 patients, there were 117 events, 52 of which were in African Americans, 45 of which were in Caucasians. Proportional hazards assumptions were verified by visual inspection of log-log plots and using a non-zero slope test of the Schoenfeld residuals [21] (p = 0.91 for African Americans, p = 0.79 for Caucasians). The method of Kaplan and Meier was used for plotting genotypes and survival, where death due to colorectal cancer was again considered as the endpoint. These graphs were generated using SigmaPlot 12.0 (Systat Software, San Jose, CA). Significance was tested using the Log-Rank method. Tests for linear trend were also conducted by including the genotype variable in the model as a continuous ordinal variable (*P*_trend_). Differences in rs4919510 genotypes in normal versus mutant KRAS tumors were assessed using Fisher's exact test.

## Results

### Study population and miR-608 genotype analysis

We investigated the relationship between rs4919510, a SNP in the mature sequence of *hsa-mir-608*, with regards to risk and disease outcome in 245 colorectal cancer cases and 446 controls. Relevant population characteristics are described in Table 1. Overall, the cases did not differ from the controls in terms of age or race; however, the case cohort contained significantly more males than each respective control group (population, hospital, or total) (Table 1). The minor allele frequency [22] of rs4919510 was significantly higher in African Americans, compared to Caucasians; 40.5% and 17.5%, respectively (Table 2). In addition, the frequency of the GG genotype was significantly higher in African Americans (15%) compared to Caucasians (3%) controls, in agreement with observations for this SNP in HapMap [23]. Since the frequencies of rs4919510 genotypes did not differ between hospital and population control groups, all analyses are presented using the overall control group (Tables 2 and 3). Rs4919510 did not deviate from Hardy-Weinberg equilibrium proportions in either the population or hospital controls.

### No association between rs4919510 and colorectal cancer risk

The GG or CG genotypes of rs4919510 were not associated with colorectal cancer risk (Table 2). In the total population, the frequency of the GG genotype was 8% in cases and 8% in controls. In African Americans, the GG genotype was represented in 15% of controls, and 13% of cases, corresponding to an adjusted OR of 0.89 (95% CI, 0.41–1.80). In Caucasians, the GG genotype was present in 3% of controls and 5% of cases, with an OR of 1.76, (95% CI, 0.48–6.39). There is no association between the SNP and KRAS mutation status in the whole cohort. In African Americans, it was notable that the GG genotype was not observed in mutant KRAS tumors, but the difference did not reach statistical significance (Figure S1).

### Association between rs4919510 and colorectal cancer survival

We found a significant association between rs4919510 and colorectal cancer survival. In Caucasians, the homozygous variant genotype, GG, was associated with a significant increase in risk of death from colorectal cancer represented by a univariable hazard ratio (HR) of 3.54 (95% CI, 1.38–9.12), which remained significant after adjustment for age, gender, histology and stage in our multivariable model; HR_GG *vs.* CC_ 2.95 (95% CI, 1.13–7.71) (Table 3) (Figure 1A). The CG genotype also showed a trend towards poor outcome, although the comparison to the CC referent genotype was not statistically significant (Table 3).

In African Americans, we observed a protective association between the GG genotype and survival, although the model approached significance in both the adjusted (HR_ GG *vs.* CC_ 0.38 [95% CI, 0.13–1.13; p = 0.082]) and non-adjusted (HR_ GG *vs.* CC_ 0.36 [95% CI, 0.12–1.07; p = 0.066]) models (Table 3) (Figure 1B). Kaplan-Meier survival curves graphically depict the divergent effects of this SNP in these populations (Figure 1). No significant survival associations were found when rs4919510 was analyzed without stratification, or when the cohort was stratified respective of cancer stage (Table 3 and Data not shown).

## Discussion

Our results show an association between a germline variant in *hsa-mir-608* with prognosis, but not risk, of colorectal cancer. In Caucasians, it was associated with an increased risk of death due to colorectal cancer. However, a trend towards the opposite effect was observed in African Americans. Previous studies have also demonstrated how race can modulate the effect of a SNP in colorectal cancer [24]. Of note however, while the association was statistically significant in Caucasians (n = 145), the association only approached significance in African Americans. This could suggest that the relationship is not epidemiologically relevant, but given the smaller number of individuals in the African American group (n = 94), it is perhaps more reflective of our reduced power. In both populations, the SNP was associated with survival in a recessive model.

While this manuscript was in preparation, Xing *et al.* published a study on colorectal cancer prognosis in association with rs4919510 within a Chinese population with 408 patients [25]. They reported that the variant allele (C) was associated with a reduced risk of recurrence-free and overall survival (HR = 0.61, 95% CI = 0.41–0.92), therefore, the G allele was associated with adverse outcome. This is in general agreement with our findings in Caucasians. Xing *et al.* did not include a control arm to examine risk associations, though as described above, our results do not support a role for this SNP in colorectal cancer. These studies strengthen the hypothesis that there is an association between rs4919510 and colorectal cancer prognosis that might be race-specific.

While we cannot currently explain the exact functional mechanism through which this SNP may affect colorectal cancer prognosis, there are several plausible hypotheses. As mentioned before, rs4919510 lies within the mature sequence of miR-608, and is located at the joint of the stem with the canonical hairpin loop. Since this rigid secondary structure is a requisite for recognition, and thus processing, of precursor miRNA by the RNAse Drosha, it is feasible that disruption of structure at this critical point might affect recognition or subsequent processing. Each miRNA has hundreds of targets, thus a singular change in a cell's miRNA kinetic profile could have an exponentially large effect on protein output, perhaps having an effect so large it could skew, even slightly, overall prognosis of a disease.

A change in a miRNA's mature sequence could also theoretically alter its target repertoire once processed [26], [27]. Although rs4919510 does not lie within the seed sequence of miR-608, hybridization kinetics outside of this region have been shown to be important in target recognition [28]. The exact importance, relative weight, and subsequent shifting of preference due to miRNA sequence variations has yet to be fully elucidated, though examples exist [12]. An extension of this principle could be applied to the SNP in *hsa-mir-608* in relation to colorectal cancer, whose putative targets include *BCL-xL*, *SEPT9*, and *CDK6* (www.targetscan.org). Indeed, an alteration in transcript targeting by miR-608 to any of those genes could have consequences directly related to cancer cell survival.

Interestingly, Xing *et al.* also reported that the association between the GG genotype and poor outcome was only observed in those patients who had been treated with chemotherapy, the majority of which had been on a FOLFOX regimen. Interestingly, miR-608 is predicted to target at least 2 of the key enzymes involved in activation of 5-FU, thymidine kinase and folylpolyglutamate synthase (www.targetscan.org). In addition, there have been several studies examining variant chemotherapeutic response rates in colorectal carcinoma by race. In one study, although time to disease progression was not affected by race, a significantly higher percentage of Caucasians responded to a FOLFOX or IROX regime [29]. Therefore, rs4919510 could theoretically play a role in differential response rates to chemotherapeutics across populations.

In addition to a putative functional association with miR-608, linkage disequilibrium with SNPs in neighboring genes cannot be ruled out. Of note, there is significantly less linkage predicted for rs4919510 from current 1000 genomes data in the African American population, where only 7 SNPs in 2 unique genes are predicted to be in linkage disequilibrium with rs4919510, as opposed to 39 SNPs in 5 unique genes in Caucasians (www.broadinstitute.org/mpg/snap/ldsearch.php#, r^2^>0.8 and distance<500 kb). Of particular interest among the list of differentially linked gene alleles in Caucasians is leucine zipper tumor suppressor 2 (*LZTS2*), which contains several SNPs in the first intron and 5′ UTR predicted to be strongly associated with rs4919510. Recent findings from two separate studies have shown that knockdown of LZTS2 expression sensitizes cells to paclitaxel therapy in addition to antagonizing proliferation of several cancer cell lines by down-regulation of myc and cyclin D1 through engagement of the NF-κB pathway [30], [31]. If the G allele of rs4919510 is truly in linkage with a SNP in *LZTS2*, the expression of this gene may be altered, contributing to the poor prognosis of this variant genotype in our Caucasian cohort. However, this hypothesis remains to be investigated.

In summary, our study validates the hypothesis that a SNP in the mature sequence of *hsa-mir-608* can significantly affect the prognosis of colorectal cancer patients. Interestingly, this effect of this SNP seems to vary by race, as demonstrated by our findings and others [25]. While the association between this SNP and colorectal cancer survival is notable as demonstrated in three cohorts, further studies should be conducted to confirm the race-specific nature of the SNP's effect. In addition, exploration of the functional role of this SNP, the mechanism of its interaction with 5-FU, as well as its potential involvement in other cancers is warranted.

## Supporting Information

Figure S1 rs4919510 and KRAS mutation status. Percentage of KRAS mutations in rs4919510 CC, CG and GG samples in the whole population A), Caucasians B), and African Americans C). Exact percentages shown in D). WT denotes wild-type, mut denotes mutant.
(PDF)

## References

[1] Siegel R, Ward E, Brawley O, Jemal A (2011). Cancer statistics, 2011: the impact of eliminating socioeconomic and racial disparities on premature cancer deaths.. CA Cancer J Clin.

[2] Alexander DD, Waterbor J, Hughes T, Funkhouser E, Grizzle W (2007). African-American and Caucasian disparities in colorectal cancer mortality and survival by data source: an epidemiologic review.. Cancer Biomark.

[3] Bartel DP (2009). MicroRNAs: target recognition and regulatory functions.. Cell.

[4] Griffiths-Jones S, Grocock RJ, van Dongen S, Bateman A, Enright AJ (2006). miRBase: microRNA sequences, targets and gene nomenclature.. Nucleic Acids Res.

[5] Guo H, Ingolia NT, Weissman JS, Bartel DP (2010). Mammalian microRNAs predominantly act to decrease target mRNA levels.. Nature.

[6] Thai TH, Christiansen PA, Tsokos GC (2010). Is there a link between dysregulated miRNA expression and disease?. Discov Med.

[7] Iorio MV, Croce CM (2009). MicroRNAs in cancer: small molecules with a huge impact.. J Clin Oncol.

[8] Schetter AJ, Leung SY, Sohn JJ, Zanetti KA, Bowman ED (2008). MicroRNA expression profiles associated with prognosis and therapeutic outcome in colon adenocarcinoma.. JAMA.

[9] Landi D, Gemignani F, Naccarati A, Pardini B, Vodicka P (2008). Polymorphisms within micro-RNA-binding sites and risk of sporadic colorectal cancer.. Carcinogenesis.

[10] Boni V, Zarate R, Villa JC, Bandres E, Gomez MA (2010). Role of primary miRNA polymorphic variants in metastatic colon cancer patients treated with 5-fluorouracil and irinotecan..

[11] Volinia S, Calin GA, Liu CG, Ambs S, Cimmino A (2006). A microRNA expression signature of human solid tumors defines cancer gene targets.. Proc Natl Acad Sci U S A.

[12] Ryan BM, Robles AI, Harris CC (2010). Genetic variation in microRNA networks: the implications for cancer research.. Nat Rev Cancer.

[13] Quach H, Barreiro LB, Laval G, Zidane N, Patin E (2009). Signatures of purifying and local positive selection in human miRNAs.. Am J Hum Genet.

[14] Yu Z, Li Z, Jolicoeur N, Zhang L, Fortin Y (2007). Aberrant allele frequencies of the SNPs located in microRNA target sites are potentially associated with human cancers.. Nucleic Acids Res.

[15] Bashyam MD, Bair R, Kim YH, Wang P, Hernandez-Boussard T (2005). Array-based comparative genomic hybridization identifies localized DNA amplifications and homozygous deletions in pancreatic cancer.. Neoplasia.

[16] Daido S, Takao S, Tamiya T, Ono Y, Terada K (2004). Loss of heterozygosity on chromosome 10q associated with malignancy and prognosis in astrocytic tumors, and discovery of novel loss regions.. Oncol Rep.

[17] Kim JH, Dhanasekaran SM, Mehra R, Tomlins SA, Gu W (2007). Integrative analysis of genomic aberrations associated with prostate cancer progression.. Cancer Res.

[18] Zheng HT, Peng ZH, Zhou CZ, Wang ZW, Qiu GQ (2005). [Refined mapping of loss of heterzygosity on 10q23-24 in sporadic colorectal carcinoma].. Zhonghua Yi Xue Za Zhi.

[19] Wang X, Zbou C, Qiu G, Fan J, Tang H (2008). Screening of new tumor suppressor genes in sporadic colorectal cancer patients.. Hepatogastroenterology.

[20] Goodman JE, Bowman ED, Chanock SJ, Alberg AJ, Harris CC (2004). Arachidonate lipoxygenase (ALOX) and cyclooxygenase (COX) polymorphisms and colon cancer risk.. Carcinogenesis.

[21] Hess KR (1995). Graphical methods for assessing violations of the proportional hazards assumption in Cox regression.. Stat Med.

[22] Mafra L, Santos SM, Siegel R, Alves I, Paz FA (2012). Packing interactions in hydrated and anhydrous forms of the antibiotic Ciprofloxacin: a solid-state NMR, X-ray diffraction, and computer simulation study.. J Am Chem Soc.

[23] Thorisson GA, Smith AV, Krishnan L, Stein LD (2005). The International HapMap Project Web site.. Genome Res.

[24] Zanetti KA, Haznadar M, Welsh JA, Robles AI, Ryan BM (2012). 3′-UTR and Functional Secretor Haplotypes in Mannose-Binding Lectin 2 Are Associated with Increased Colon Cancer Risk in African Americans.. Cancer Res.

[25] Xing JL, Wan S, Zhou F, Qu F, Li B (2011). Genetic polymorphisms in pre-microRNA genes as prognostic markers of colorectal cancer..

[26] Kawahara Y, Zinshteyn B, Sethupathy P, Iizasa H, Hatzigeorgiou AG (2007). Redirection of silencing targets by adenosine-to-inosine editing of miRNAs.. Science.

[27] Jazdzewski K, Murray EL, Franssila K, Jarzab B, Schoenberg DR (2008). Common SNP in pre-miR-146a decreases mature miR expression and predisposes to papillary thyroid carcinoma.. Proc Natl Acad Sci U S A.

[28] Mishra PJ, Humeniuk R, Longo-Sorbello GS, Banerjee D, Bertino JR (2007). A miR-24 microRNA binding-site polymorphism in dihydrofolate reductase gene leads to methotrexate resistance.. Proc Natl Acad Sci U S A.

[29] Sanoff HK, Sargent DJ, Green EM, McLeod HL, Goldberg RM (2009). Racial differences in advanced colorectal cancer outcomes and pharmacogenetics: a subgroup analysis of a large randomized clinical trial.. J Clin Oncol.

[30] Ji D, Deeds SL, Weinstein EJ (2007). A screen of shRNAs targeting tumor suppressor genes to identify factors involved in A549 paclitaxel sensitivity.. Oncol Rep.

[31] Kim JM, Song JS, Cho HH, Shin KK, Bae YC (2011). Effect of the modulation of leucine zipper tumor suppressor 2 expression on proliferation of various cancer cells functions as a tumor suppressor.. Mol Cell Biochem.

