# Supplementary figures and images for: rs4919510 in hsa-mir-608 Is Associated with Outcome but Not Risk of Colorectal Cancer
Source: PLoS One. 2012 May 11;7(5):e36306. doi: 10.1371/journal.pone.0036306 (PMC3350523; doi:10.1371/journal.pone.0036306)

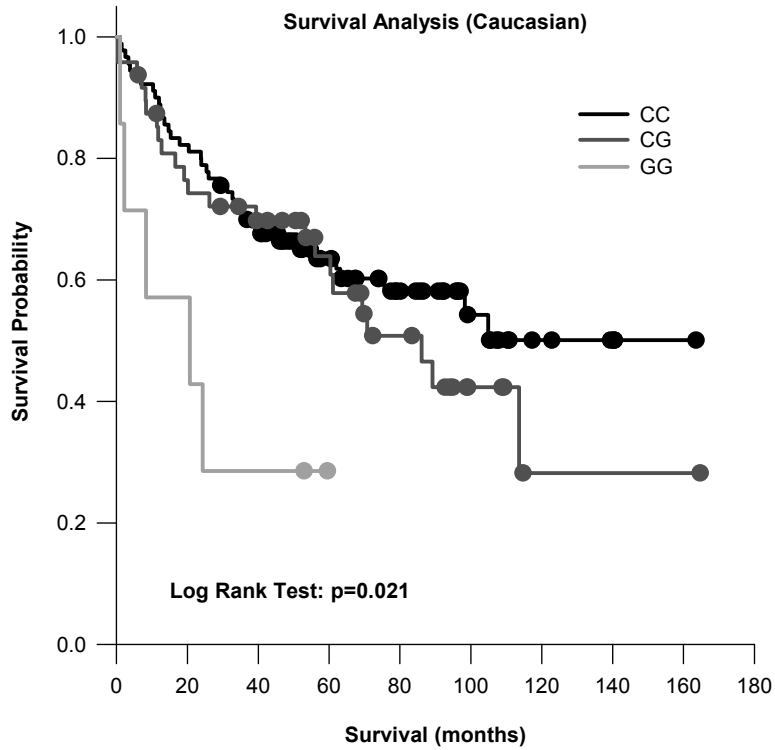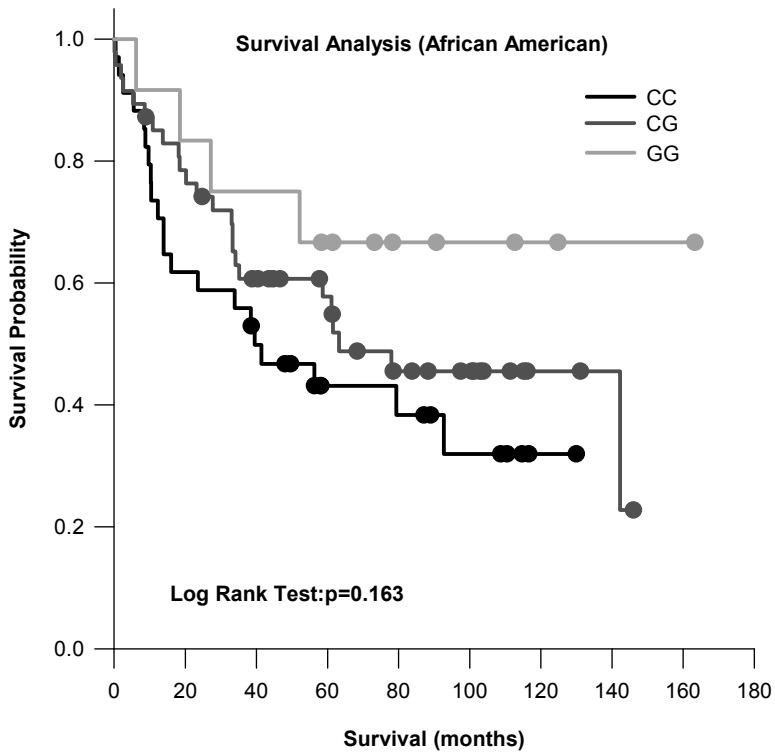

Supplement: Figure S1 — rs4919510 and KRAS mutation status. Percentage of KRAS mutations in rs4919510 CC, CG and GG samples in the whole population A), Caucasians B), and African Americans C). Exact percentages shown in D). WT denotes wild-type, mut denotes mutant. (PDF) [file pone.0036306.s001.pdf]
